# An Overview of the Clinical Use of Antimuscarinics in the Treatment of Overactive Bladder

**DOI:** 10.1155/2011/820816

**Published:** 2011-06-07

**Authors:** Anastasios Athanasopoulos, Konstantinos Giannitsas

**Affiliations:** Urodynamic Urology Unit, Department of Urology, Medical School, University of Patras, 26500 Patra, Greece

## Abstract

Overactive bladder is a common and bothersome condition. Antimuscarinic agents, as a class, are the cornerstone of medical treatment of overactive bladder. They offer significant improvements in symptoms and patients' quality of life. Antimuscarinics are generally well tolerated with mild and predictable side effects. Available antimuscarinics have small, yet statistically significant, differences in their efficacy and tolerability profiles. In clinical practice, finding the agent that offers the optimum balance of efficacy and side effects for an individual patient remains the major challenge.

## 1. Introduction

Overactive bladder (OAB) is a lower urinary tract condition, characterized by symptoms of urgency, with or without urge incontinence, usually with frequency and nocturia. This symptom complex significantly affects patient's quality of life. In most cases, the underlying pathophysiology is an involuntary detrusor contraction during the storage phase of the voiding cycle [1].

Normal bladder contraction during voiding involves stimulation of the muscarinic receptors on the detrusor muscle by acetylcholine (Ach). The role of Ach in the pathogenesis of involuntary contractions during the storage phase is elusive. Despite the fact that detrusor may contract spontaneously, as a result of the intrinsic activity of the myocyte or of small units of smooth muscle cells [2], Ach is still released from the nerves or from nonneurogenic sources, like the urothelium. A direct or indirect effect of Ach should be considered [2]. Muscarinic receptors are also found on the presynaptic nerve terminals to the bladder participating in the regulation of transmitter release. Currently, there is increasing evidence for an important role of afferent pathways in the pathophysiology of involuntary detrusor contractions [2, 3]. Antimuscarinics, which block muscarinic receptors, have been the treatment of choice for overactive bladder for decades. They affect the efferent control on detrusor contraction, but increasing evidence also suggests a role in afferent pathways' regulation. 

There are several subtypes of muscarinic receptors. The human detrusor contains mainly the M_2_ and M_3_ subtypes [4]. Available antimuscarinics vary in their selectivity for muscarinic receptors.

 Oxybutynin [5–8], Tolterodine [9], propiverine [10], solifenacin [11, 12], darifenacin [13, 14], trospium [15, 16], and fesoterodine [17] are antimuscarinic agents approved for use in OAB treatment. Evidence on the efficacy and safety of these agents as a class is reviewed here, and issues concerning their clinical application are discussed. 

## 2. Materials and Methods

The current literature on the efficacy and safety of antimuscarinics was reviewed by searching Medline/PubMed for relevant articles, published in English between 1980 and 2010. 

## 3. Results and Discussion

### 3.1. Antimuscarinics

#### 3.1.1. Oxybutynin

Oxybutynin is the first antimuscarinic used for the treatment of OAB. In addition to its antimuscarinic action, oxybutynin in high doses exerts muscle-relaxant and local anaesthetic effects [5–8].

 Oxybutynin is now available in oral, immediate (IR) and extended release (ER), as well as two transdermal formulations, a patch and a gel. An intravesical formulation of oxybutynin has also been studied [18].

Oxybutynin IR formulation was the fist that entered clinical practice. Despite its satisfactory efficacy, the substantial incidence of dry mouth, immediate release oxybutynin's most common and bothersome side-effect, limited its tolerability. Newer formulations aimed at eliminating peaks in concentration of oxybutynin and its metabolites in order to reduce related side effects. 

The ER formulation of oxybutynin provides a smooth plasma concentration profile over the 24-hour dosage interval, facilitating once-daily administration. Hence, given its overall efficacy/tolerability and dose flexibility, oxybutynin ER provides an alternative in the first line of pharmacotherapy for OAB [8]. Overall, as shown in the OPERA study [19], oxybutynin ER has modestly greater efficacy than tolterodine ER at its most commonly prescribed dose. In the OBJECT study, oxybutynin ER was more effective than tolterodine IR at the endpoints of urge incontinence, total incontinence, and micturition frequency episodes [20].

The transdermal oxybutynin (OXY-TDS) formulation offers patients with urinary incontinence an effective, safe and well-tolerated option for managing the symptoms of overactive bladder [7, 21]. As OAB contributes to decreased work productivity due to job interruptions as well as fatigue, the use of OXY-TDS may result in productivity improvement when patients receive 3.9 mg/day via twice weekly patch application for up to 6 months [22].

Oxybutynin chloride topical gel (OTG) was approved in January 2009 by the US FDA. OTG was designed to provide steady plasma oxybutynin levels with daily application, favorably altering the circulating N-desethyloxybutynin metabolite to oxybutynin ratio, thus minimizing the antimuscarinic adverse effects of oral formulations. The use of a biocompatible delivery system also reduced the application-site skin reactions associated with other available forms of transdermal delivery. OTG represents an efficacious, safe, and convenient alternative to other oxybutynin formulations and oral antimuscarinics for the treatment of OAB [23].

Interestingly, all the above-mentioned oxybutynin formulations have been shown to be more efficacious than the IR oxybutynin [24, 25] in respective trials. 

#### 3.1.2. Tolterodine

Tolderodine is a widely prescribed antimuscarinic and, it was the first specifically developed to treat OAB. Tolterodine is not selective for any muscarinic receptor subtype, but it exhibits selectivity for the urinary bladder over salivary glands in vivo [26].

An IR formulation was available first, but an ER, administered once daily, formulation was later designed. Its efficacy and tolerability have been proved in a large number of trials [27]. Tolterodine offers significant improvement in overactive bladder symptoms and quality of life while having a favorable safety profile. It soon became the gold standard in the class, a drug that all others are compared to, during their clinical development.

Oxybutynin and tolterodine, the until relatively recent years most commonly prescribed antimuscarinics, have been shown to have similar efficacies in general OAB populations [28], as well as in specific subpopulations defined by severity of urodynamic findings [29]. 

#### 3.1.3. Propiverine

Propiverine, another muscarinic receptor antagonist, has also been demonstrated to inhibit L-type Ca^++^ channels in high concentrations [30].

Propiverine has similar efficacy to oxybutynin and tolterodine, similar tolerability and impact on quality of life to tolterodine, but a better tolerability profile than oxybutynin [31, 32]. This drug is well tolerated [33]. 

Propiverine and oxybutynin are efficacious in children with incontinence due to overactive bladder and propiverine is officially approved in certain countries for pediatric use. Alloussi et al. [34] evaluated existing evidence for the use of antimuscarinics in children. They concluded that high-quality studies are still limited and results vary widely across antimuscarinics. This fact is associated with different levels of evidence and grades of recommendation for children for oxybutynin (3 C), propiverine (1 B/C), tolterodine (3 C), and trospium chloride (3 C), awarded by the International Consultation on Incontinence. The daily urgency episodes were significantly reduced from baseline to 12 weeks on propiverine treatment, compared with placebo. Secondary endpoints, including sum of urgency severity per 24 h, urgency severity per void, and daytime voiding frequency, were also improved significantly in the propiverine group [35]. 

#### 3.1.4. Darifenacin

Darifenacin is the antimuscarinic with the highest M-3 receptor subtype selectivity. Long-term darifenacin treatment was associated with significant and clinically meaningful improvements in quality of life of patients with urge incontinence (“wet” OAB) over 2 years [36]. In a study of patients who were dissatisfied with their previous treatment with oxybutynin ER or tolderodine ER, patients perception of bladder condition (PPBC) score and OAB symptoms were significantly improved, and satisfaction was high during treatment with darifenacin 7.5 or 15 mg [37]. Haab [14] in a comprehensive review described the good clinical efficacy and safety profile of this agent. 

#### 3.1.5. Solifenacin

A pooled analysis of four randomized, placebo-control1ed, phase III studies of solifenacin in OAB patients without incontinence, showed a significant improvement of symptoms and voided volume after 12 weeks of treatment [38]. 

How does solifenacin compare to longer established antimuscarinics? One comparison of the “new” (solifenacin and darifenacin) and “old” antimuscarinic agents showed the two generations of treatment had similar efficacy [35, 39]. A randomized, double-blind study found that solifenacin is superior to an encapsulated formulation of tolterodine ER in most of the efficacy outcomes [40]. The majority of side effects were mild to moderate in nature, yet significantly more for solifenacin, and discontinuations were comparable and low in both groups. This study investigated both approved doses of solifenacin, 5 mg and 10 mg, and was, therefore, criticized for using doses not directly comparable to tolterodine 4 mg. A subanalysis of this study [30] subsequent compared solifenacin 5 mg and tolderodine 4 mg and better reflected treatment outcomes with the doses most commonly used in clinical practice, at least during treatment initiation. It concluded that after a 4-weeks treatment period, solifenacin 5 mg significantly improved incontinence symptoms and reduced the use of incontinent pads, compared to tolterodine. In another, randomized, placebo-controlled study, Cardozo et al. [41] found that solifenacin significantly reduced the number of urgency episodes and urgency bother and was well tolerated. Treatment was effective as early as day 3.

Solifenacin is the first antimuscarinic to demonstrate significant warning-time improvement in a large OAB clinical trial conducted to evaluate warning time and diary variables in the same study population [42]. 

A relatively recent comprehensive review for solifenacin concluded that this agent was effective in the treatment of OAB with urge incontinence [12]. 

#### 3.1.6. Trospium

Trospium chloride is a quaternary ammonium compound. It does not cross the blood-brain barrier; therefore, no central nervous system adverse events are anticipated [16]. This drug significantly reduces UUI and frequency compared with placebo [43]. Compared to tolderodine, trospium reduced the frequency of micturition and incontinence episodes. Extended-release trospium chloride 60 mg, a novel modified-release form of this compound allows once-daily administration, potentially enhancing compliance to treatment and improving its clinical efficacy/tolerability profile, compared with immediate-release form [44]. Cardozo et al. [44] in a recent publication underlined that the extent of metabolism of this drug is low and independent of the liver cytochrome P450 isoenzyme system. This pharmacodynamic profile further simplifies decision making in polypharmacy situations, such as multimorbid and elderly patients. Furthermore, subject to predominantly renal elimination as the unchanged form, trospium chloride retains its pharmacological activity within the urinary bladder, and local action on urothelium muscarinic receptors is supposed to contribute to its early onset and sustained efficacy in controlling urgency. 

#### 3.1.7. Fesoterodine

Fesoterodine is the newest antimuscarinic for the treatment of OAB. Fesoterodine is a prodrug. It is rapidly and extensively hydrolyzed by nonspecific esterases, thus bypassing the CYP system, to 5-hydroxymethyl tolderodine (5-HMT), which is also the active metabolite of tolderodine. Interestingly, as 5-HMT formation from fesoterodine occurs via ubiquitous nonspecific esterases, the rate of fesoterodine hydrolyzation maybe more uniform and complete.

Initial data from phase 2 trials showed that fesoterodine was an effective and well-tolerated therapy for OAB [45]. In subsequent clinical studies, fesoterodine doses of 4 and 8 mg/day were consistently superior to placebo in improving overactive bladder symptoms, with 8 mg/day having significantly greater effects than 4 mg/day [17]. Both doses were safe and well tolerated, with a low overall incidence of adverse events. Tolerability is comparable to that of tolderodine (ER) [46]. 

In a posthoc analysis of pooled data from two clinical trials including 1,548 women with overactive bladder, fesoterodine 4 mg and 8 mg and tolderodine showed significant improvements in all bladder diary variables assessed and greater response rates versus placebo. Fesoterodine 8 mg was significantly more efficacious than fesoterodine 4 mg and tolderodine ER in improving UUI episodes and continence days per week [47]. Recently, the FACT study, a head to head placebo controlled trial, compared the efficacy and tolerability of fesoterodine 8 mg with tolderodine ER 4 mg. This study was designed to asses the superiority of fesoterodine over tolderodine ER for the treatment of OAB symptoms, and 1697 patients were included. This trial concluded that in patients with OAB, fesoterodine 8 mg showed superior efficacy over tolderodine ER 4 mg and placebo in reducing UUI episodes and in improving most patient-reported outcome measures. Both active treatments were well tolerated [48]. In another recent study, the flexible dose of fesoterodine was evaluated. Among 516 subjects treated, approximately 50% opted for dose escalation to 8 mg at week 4. The study concluded that flexible dose fesoterodine significantly improved OAB symptoms health related quality of life (HRQOL) and rates of treatment satisfaction and was well tolerated in patients with OAB who were dissatisfied with prior tolderodine therapy [49]. 

### 3.2. Efficacy and Safety

Currently available antimuscarinics have all demonstrated their efficacy and safety in well-designed, controlled studies conducted during their clinical development. The significant placebo effect observed in OAB trials and the frequent treatment discontinuations in real-life practice have often raised doubts regarding the true efficacy and/or safety of this drug class. Systematic reviews and meta-analyses of existing data have tried to clarify uncertainties.

In 2003, Herbison et al. [39] have published a systematic review of randomized controlled trials comparing antimuscarinic agents to placebo in the treatment of OAB. The authors concluded that antimuscarinic drug therapy provided significant improvement in OAB symptoms, such as urge incontinence episodes and micturition frequency over placebo. Significant improvements, compared to placebo have also been demonstrated for urodynamic parameters. However, the magnitude of treatment effect was smaller than the anticipated based on clinical experience with antimuscarinics. A possible explanation for this is the common combination of medical treatment and bladder training in clinical practice. In the majority of clinical trials, formal bladder training is not included. 

A 2005 systematic review of 52 randomized, controlled trials by Chapple et al. [50], which was latter updated with more studies in 2008 [51], showed that antimuscarinics, as a class, significantly reduce urge incontinence episodes, making many patients continent, and provide significant improvements in quality of life. They also reduce the severity of urgency and decrease micturition frequency. Individual antimuscarinics were effective in at least one of the outcome measures included in the reviews. Profiles of each drug and dosage differ and should be considered in making treatment choices. Despite abundance of evidence on the short-term efficacy of antimuscarinics, Chapple et al. noted a lack of knowledge regarding issues of chronic treatment, given that followup in most trials is short. 

Differences in efficacy of antimuscarinics have often reached statistical significance in clinical trials. Nevertheless, the magnitude of these differences is not readily appreciated in everyday clinical practice and many clinicians consider drugs in this class as “comparable” in terms of efficacy. According to a meta-analysis by Novara et al. [24], efficacies of available antimuscarinics are comparable. Nevertheless, if factors such as safety, tolerability, and cost are to be taken into account, Oxybutynin ER, tolterodine ER 4 mg, solifenacin 5 mg, or solifenacin 10 mg can be considered the first-line treatment choice. Darifenacin 15 mg and fesoterodine 4 mg are alternatives although more data are needed. In cases of lack of efficacy of first-line ER drug, fesoterodine 8 mg and solifenacin 10 mg might be second-line treatment, given that their efficacy is superior with only a small compromise in tolerability.

As far as safety is concerned, antimuscarinics, in general, are safe [24, 52, 53]. The “older” drugs oxybutynin and tolterodine have been more thoroughly studied [54, 55]. Side effects are due to muscarinic receptor binding in organs other than the bladder. The effects of antimuscarinics on salivary glands are responsible for the most common and bothersome side effect, dry mouth. Other unwanted effects include constipation, blurred vision, somnolence, dizziness and cognitive impairment. Untreated, close-angle glaucoma is a contraindication for antimuscarinics.

Slight differences in the safety profiles of existing antimuscarinic agents depend on their selectivity for specific muscarinic receptor subtypes, selectivity for the bladder compared to salivary glands, their lipophilicity and ability to cross the blood-brain barrier, as well as their pharmacokinetic properties. 

Evidence from controlled trials [50] suggests that antimuscarinics are well tolerated compared with placebo, with the exception of immediate-release (IR) oxybutynin. Extended-release (ER) tolterodine is the only formulation with fewer total treatment discontinuations compared with placebo, a finding that just reached statistical significance.

In general, the extended release formulations are better tolerated than the immediate release ones. In cases that dry mouth is intolerable with the oral formulations, transdermal oxybutynin might bean alternative, but, unfortunately, application site reactions are common with the oxybutynin patch [21]. Solifenacin and darifenacin are believed to be associated with higher rates of constipation [24] compared to other antimuscarinics. Nevertheless, a recent meta-analysis of randomized, placebo-controlled trials has shown high odds ratios for constipation, compared with placebo, for other drugs as well [56].

Central nervous system side effects are a concern when prescribing antimuscarinics for the treatment of OAB, particularly in vulnerable populations such as the elderly and CNS-compromised, neurogenic bladder patients. The evidence for cognitive impairment with oxybutynin is compelling [57]. Darifenacin with low CNS penetration and selectivity for the M3 over the M1 muscarinic receptor subtype is expected to cause less cognitive impairment. Indeed, in a short term study, darifenacin did not have any effect on the cognitive function [58]. Moreover, a review of available literature [59] concluded that darifenacin did not cause an impairment of memory among other cognitive functions. Trospium chloride is another drug that does not cross the blood-brain barrier and in a recent study it was undetectable in the older human central nervous system [60]. Fesoterodine is considerably less lipophilic than tolderodine [61] which has minimal or no cognitive effects.

The binding of muscarinic receptors in the heart may lead to cardiovascular adverse events and QT interval prolongation has been a concern with antimuscarinics. In a randomized, double-blind, placebo-controlled, crossover trial [62], tolterodine significantly increased heart rate versus placebo and darifenacin did not affect heart rate compared to placebo. Darifenacin does not prolong QT/QTc interval [63]. An older study showed that Oxybutynin is not associated with a corrected QT interval prolongation and is unlikely to induce ventricular arrhythmias [64]. It seems that tolterodine does not have a clinically significant effect on QT interval [65]. Propiverine provoked a statistically significant increase in the mean QTc interval but without clinical arrhythmic events [66]. The newest antimuscarinic drug fesoterodine is not associated with QTc prolongation or other ECG abnormalities at either therapeutic or supra-therapeutic doses [67]. In a study with real-life conditions, that is, with inclusion of large numbers of patients with cardiovascular comorbidities and taking several other medications, therapeutically effective doses of solifenacin did not increase heart rate or blood pressure [68]. 

### 3.3. Special Treatment Issues

#### 3.3.1. Treatment Compliance

Despite acceptable rates of treatment discontinuation in clinical trials, real-life compliance, especially to long-term treatment, is low. For example, in a pharmacy dispensing records review for antimuscarinic agents from January 2003 to December 2006 conducted for the United States Military Health System National Capital Region, 35% of OAB patients did not refill a fully reimbursed prescription for antimuscarinics [69]. In another study, 44.5% of patients did not renew their first prescription of antimuscarinics [70]. In observational trials, under real-life conditions, discontinuation rates for tolterodine, for example, have been reported to be as high as 49% at 6 months followup [71]. Overall, adherence is significantly better for extended release than immediate release agents.

Low compliance to treatment can be due to inadequate drug efficacy, intolerable side effects, poor patient education and follow up, and cost issues. Among these reasons dry mouth is the most common [51, 72]. In every-day clinical practice, unmet expectations may represent another important reason for treatment discontinuation [73, 74]. Selecting the appropriate drug for each patient, the one that offers the best balance between efficacy and adverse events would be a very important step in improving adherence to treatment. Patient education on OAB and its treatments and patient reassurance when side effects occur represent other important strategies in improving compliance. Realistic patient expectations from treatment are a prerequisite for treatment success. 

#### 3.3.2. Dose Flexibility

Dose flexibility offers the advantage of an individually tailored treatment to achieve the optimum balance between efficacy and adverse events. A strategy based on patient-requested dose increases has been found to consistently improve overactive bladder symptoms. The impact of dose flexibility on clinical management of OAB has been examined in studies with solifenacin, darifenacin, and oxybutynin ER [75]. Patients requesting a dose increase usually had more severe symptoms at baseline than those who did not request uptitration. Patients with severe symptoms at baseline benefit more from the increased dose [75]. In clinical trials, about 50% of the patients ask for an increase of the dose of their medication [40, 49]. Selecting a drug which offers dose flexibility seams a reasonable first-line treatment approach. 

#### 3.3.3. Switch between Antimuscarinics

Despite the fact that differences in efficacy of antimuscarinics in clinical trials with large OAB populations are relatively small, an individual patient may benefit more from a particular drug than another. “Salvaging” nonresponders to one drug with another has been shown in several studies. Solifenacin, for example, has been shown to significantly improve bladder diary and validated quality-of-life outcomes in women with urge incontinence that failed to respond or were unable to tolerate oxybutynin IR [75]. Solifenacin treatment in patients with residual urgency after an at-least-four-week course of tolterodine ER 4 mg was associated with significant improvements in urgency and other diary-documented symptoms of OAB. Patients treated with solifenacin also had significant improvements in quality of life scores and the perceived bother of OAB [76]. In another study, patient's perception of bladder condition (PPBC) score and OAB symptoms were significantly improved, and satisfaction was high during treatment with darifenacin (7.5/15 mg) in patients who were dissatisfied with the previous oxybutynin ER or tolterodine ER treatment [37]. In a recent study [77], patients dissatisfied with tolterodine received fesoterodine 4 mg or 8 mg. PPBC, urgency perception scale and the overactive bladder questionnaire (OAB-q) were significantly improved after 12 weeks of fesoterodine and 80% of patients became satisfied.

The above studies, despite limitations in patient selection and overall design, suggest that switch between drugs is a reasonable approach in patient failing initial treatment. 

#### 3.3.4. Safety in the Male Population

A significant concern when antimuscarinics are considered in male patients with storage lower urinary tract symptoms (LUTS), suggestive of an overactive bladder, is the risk of urinary retention. This concern seems particularly relevant when voiding, benign prostate hyperplasia-related LUTS are present. The reality, nevertheless, is that antimuscarinics at clinically recommended doses have little effect on voiding pressures. Clinical experience has proved that concerns regarding acute urinary retention or increased residual volume are unfounded [78–83]. The incidence of urinary retention is minimal (<1%), even in male patients with bladder outflow obstruction, despite the occasional increase in residual urine in certain patients. On the other hand, there are no established criteria for excluding patients at risk for retention from antimuscarinic therapy [81]. More studies with large number of patients, including patients with severe obstruction, are required. In the meantime, it seems reasonable to offer antimuscarinics, either as monotherapy or in combination with *α*-blockers to men with mild-to-moderate obstruction and small residual volumes. The addition of an antimuscarinic to a-blockers has been shown to significantly improve symptoms and quality of life of these patients [79, 81, 84, 85]. 

## 4. Conclusions

Antimuscarinic agents, as a class, are the cornerstone of medical treatment of overactive bladder. Accumulated evidence from clinical trials and meta-analyses has proved their efficacy and safety. Antimuscarinics offer significant improvements in symptoms of urge incontinence, urgency, frequency, and nocturia. This translates in substantial benefits in quality of life. Antimuscarinics are generally well tolerated with mild and predictable antimuscarinic side effects. The most common and bothersome of them, dry-mouth, not infrequently leads to treatment discontinuation.

Available antimuscarinics have small, yet statistically significant, differences in their efficacy and tolerability profiles. In general, the higher doses of drugs that offer dose flexibility have higher efficacy. Tolerability depends mainly on drug selectivity for the bladder over other organs, selectivity for muscarinic receptor subtypes and ability to penetrate the CNS. Given that there is no “cure” for OAB yet, finding the agent that offers the optimum balance of efficacy and side effects for an individual patient remains the major challenge in OAB treatment. 
